# The ATP Transporter VNUT Mediates Induction of Dectin-1-Triggered *Candida* Nociception

**DOI:** 10.1016/j.isci.2018.08.007

**Published:** 2018-08-15

**Authors:** Kenta Maruyama, Yasunori Takayama, Erika Sugisawa, Yu Yamanoi, Takashi Yokawa, Takeshi Kondo, Ken-ichi Ishibashi, Bikash Ranjan Sahoo, Naoki Takemura, Yuki Mori, Hisashi Kanemaru, Yutaro Kumagai, Mikaël M. Martino, Yoshichika Yoshioka, Hisao Nishijo, Hiroki Tanaka, Atsushi Sasaki, Naohito Ohno, Yoichiro Iwakura, Yoshinori Moriyama, Masatoshi Nomura, Shizuo Akira, Makoto Tominaga

**Affiliations:** 1 Laboratory of Host Defense, Osaka University, Osaka 565-0871, Japan; 2 Laboratory of Biofunctional Imaging, Osaka University, Osaka 565-0871, Japan; 3 WPI Immunology Frontier Research Center (IFReC), Osaka University, Osaka 565-0871, Japan; 4 Thermal Biology group, Exploratory Research Center on Life and Living Systems National Institutes of Natural Sciences, Okazaki Aichi 444-8787, Japan; 5 Division of Cell Signaling, National Institute for Physiological Sciences, National Institutes of Natural Sciences, Okazaki, Aichi 444-8787, Japan; 6 Department of Physiological Sciences, the Graduate University for Advanced Studies, Aichi 444-8787, Japan; 7 Research Laboratory, Ikedamohando Co., Ltd., 2-16-16 Iwamoto-cho, Chiyoda-ku, Tokyo 101-0032, Japan; 8 BioView Corporation, 2-16-16 Iwamoto-cho, Chiyoda-ku, Tokyo 101-0032, Japan; 9 Laboratory for Immunopharmacology of Microbial Products, School of Pharmacy, Tokyo University of Pharmacy and Life Sciences, 1432-1 Horinouchi, Hachioji, Tokyo 192-0392, Japan; 10 Department of Mucosal Immunology, School of Medicine, Chiba University, 1-8-1 Inohana, Chuou-ku, Chiba 260-8670, Japan; 11 Biotechnology Research Institute for Drug Discovery National Institute of Advanced Industrial Science and Technology Central 5-41, 1-1-1 Higashi, Tsukuba, Ibaraki 305-8565, Japan; 12 European Molecular Biology Laboratory Australia, Australian Regenerative Medicine Institute, Innovation Walk, Monash University, Wellington Road, Clayton, VIC 3800, Australia; 13 System Emotional Science (Physiology), Graduate School of Medicine and Pharmaceutical Sciences for Research, University of Toyama, 2630 Sugitani, Toyama 930-0194, Japan; 14 Research Unit/Neuroscience Sohyaku, Innovative Research Division, Mitsubishi Tanabe Pharma Corporation, 1000, Kamoshida-cho, Aoba-ku, Yokohama 227-0033, Japan; 15 Research Institute for Biomedical Sciences, Tokyo University of Science, 2669 Yamazaki, Noda, Chiba 278-0022, Japan; 16 Department of Membrane Biochemistry, Okayama University Graduate School of Medicine, Dentistry and Pharmaceutical Sciences, Okayama 700-8530, Japan; 17 Department of Medicine and Bioregulatory Science, Kyushu University, Fukuoka 812-8582, Japan; 18 Institute for Environmental and Gender Specific Medicine, Juntendo University, 2-1-1 Tomioka, Urayasu, Chiba 279-0021, Japan

**Keywords:** Molecular Mechanism of Behavior, Molecular Neuroscience, Medical Microbiology

## Abstract

*Candida albicans* infection can cause skin, vulvar, or oral pain. Despite the obvious algesic activity of *C*. *albicans*, the molecular mechanisms of fungal nociception remain largely unknown. Here we show that the *C*. *albicans*-specific signaling pathway led to severe mechanical allodynia. We discovered that *C*. *albicans*-derived β-glucan stimulated nociceptors depending on Dectin-1, and two pathways in inflammatory pain. The major pathway operates via the Dectin-1-mediated ATP-P2X_3_/P2X_2/3_ axis through intercellular relationships between keratinocytes and primary sensory neurons, which depends on the ATP transporter vesicular nucleotide transporter (VNUT). The other pathway operates via the Dectin-1-mediated PLC-TRPV1/TRPA1 axis in primary sensory neurons. Intriguingly, *C*. *albicans*-derived β-glucan has the ability to enhance histamine-independent pruritus, and VNUT inhibitor clodronate can be used to treat unpleasant feelings induced by β-glucan. Collectively, this is the first report to indicate that Dectin-1 and VNUT mediated innate sensory mechanisms that detect fungal infection.

## Highlights

• β-Glucan-induced pain is abolished in Dectin-1- and TRPV1/TRPA1-deficient mice
• β-–Glucan stimulates nociceptors via Dectin-1-PLC axis, activating neurons
• β-Glucan-induced allodynia is dependent on extracellular ATP and VNUT
• VNUT inhibitor clodronate can be used to treat β-glucan-induced allodynia

## Graphical Abstract

**Figure:**
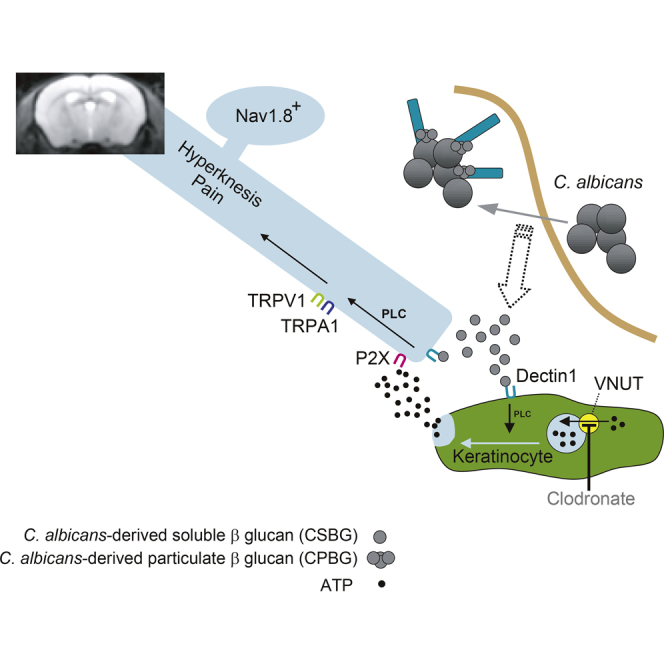

## Introduction

*Candida albicans* is an opportunistic fungus that thrives in the skin, mouth, vagina, and nipples. Immunodeficiency within hosts and poorly controlled diabetes have increased the rates of invasive *C*. *albicans* infections, evoking skin or oral pain. Notably, approximately 75% of women experience vulvovaginal candidiasis at some point during their lifetime and 5% of women experience recurrent episodes of infection (Egan and Lipsky, 2000). It is well established that such mucosal invasion of *C*. *albicans* induces mechanical allodynia and itching (Farmer et al., 2011). For example, nipples are a common site of *C*. *albicans* infection, and breast candidiasis in a lactating mother is characterized by severe nipple pain (Amir et al., 2013). It has been well established that ion channels expressed in primary sensory neurons play a critical role in the sensing of pain (Woolf and Costigan, 1999). Transient receptor potential cation channel subfamily vanilloid member 1 (TRPV1) and transient receptor potential cation channel subfamily ankyrin member 1 (TRPA1) are vital ion channels that mediate nociceptive signaling (Julius, 2013). A recent report suggested that nociceptors directly sense Gram-positive bacterial components such as α-hemolysin (Chiu et al., 2013). Another group reported that Gram-negative bacterial components, such as lipopolysaccharide, are sensed by TRPA1 (Meseguer et al., 2014). Thus, nociceptors may directly sense bacterial infection like innate immune cells.

From inside to outside, the *C*. *albicans* cell wall is composed of β-glucan and mannan (Gow et al., 2012). In response to invading fungi, innate immune cells recognize fungal surface mannan through Toll-like receptor (TLR) 4, leading to the production of cytokines via the activation of adaptor protein MyD88 and TRIF (Underhill and Lliev, 2014). A recent report suggested that mannan is also detected by TLR2, mannose receptor, Dectin-2, DC-SIGN, and Mincle (Lionakis and Netea, 2013). During the budding growth phase, β-glucan is exposed to the fungal surface and is sensed by Dectin-1 (Saijo et al., 2007). Ligand-stimulated Dectin-1 assembles a multimeric complex and induces signaling via the ITAM-like motif, leading to the activation of the CARD-9-Bcl-10-Malt-1 trimer (CBM trimer) and the NLRP3-ASC-ICE complex (NLRP3 inflammasome). Activation of the CBM trimer and the NLRP3 inflammasome is indispensable for the induction of nuclear factor (NF)-κB-dependent pro-inflammatory cytokine production and interleukin (IL)-1β maturation, respectively (Underhill and Lliev, 2014). Recently, the first fungal cytolytic peptide, named candidalysin, was discovered (Moyes et al., 2016). Because candidalysin is secreted from *C*. *albicans* and permeabilizes the epithelial membrane, it may contribute to the pathogenesis of fungal inflammation. Recently, our group discovered that *C*. *albicans* stimulates nociceptors via the β-glucan receptor Dectin-1 to induce Calcitonin gene-related peptide (CGRP). Notably, nociceptor-derived CGRP suppressed β-glucan-induced inflammation and osteoclast multinucleation via Jdp2-mediated NF-κB repression and inhibition of actin polymerization, respectively (Maruyama et al., 2017). Thus, nociceptors may modulate the fungal osteomyelitis, but mechanisms by which they sense and feel fungal invaders remains largely unknown.

In this study, we noticed that Dectin-1-deficient mice were unresponsive to fungal pain. *C*. *albicans*-derived soluble β-glucan (CSBG) induces robust pain via the Dectin-1-mediated ATP-P2X_3_/P2X_2/3_ axis and Dectin-1-mediated phospholipase C (PLC)-TRPV1/TRPA1 axis. Furthermore, CSBG has the ability to enhance Mrgpr ligand chloroquine (CQ)-induced itch behaviors. Strikingly, inhibition of the ATP transporter vesicular nucleotide transporter (VNUT) by using clodronate abolished the unpleasant feelings induced by β-glucan.

## Results

### *C*. *albicans*-Derived Soluble β-Glucan (CSBG) Is a Critical Irritant Released from *C*. *albicans*

**Figure 1:**
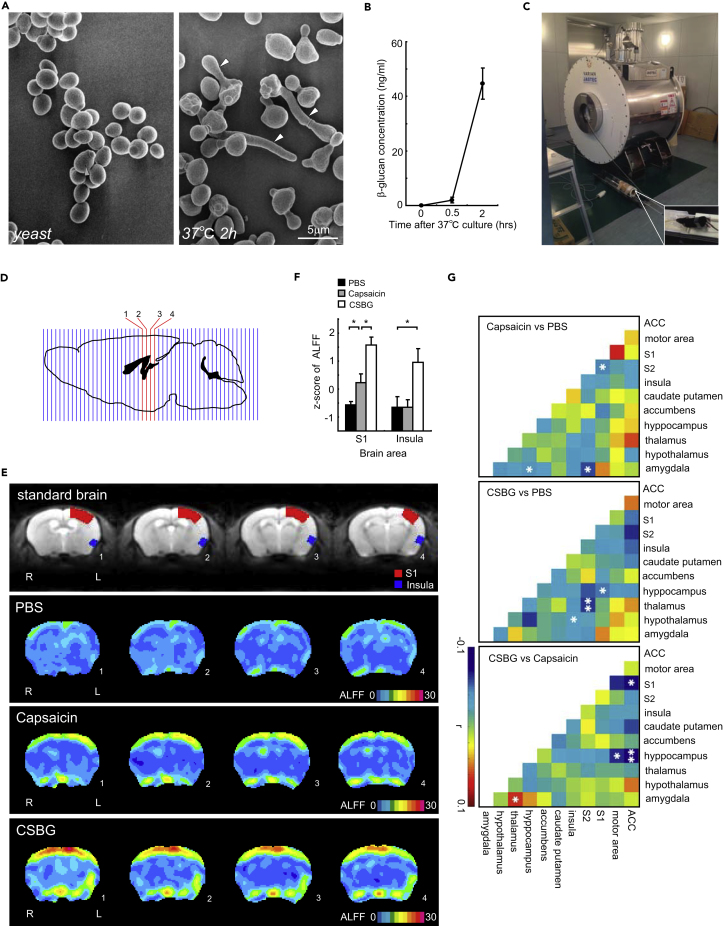
fMRI Analysis of *C*. *albicans*-Induced Pain Sensation (A and B) Morphology (A, white arrowheads: hyphae) and soluble β-glucan concentration (B, n = 4) in yeast form *C*. *albicans* cultured at 37°C. (C) Mouse MRI system. Mice were maintained under light anesthesia. phosphate buffered saline (PBS), capsaicin, or CSBG were injected into the right hind paw of mice, and fMRI images were recorded (30 min after injection, n = 6/group). (D–G) A total of 55 sections of the brain were analyzed by MRI (D). Averaged ALFF maps of sections 1–4 in (D) are displayed (E). Group average z-scores of ALFF in the S1 and insula were calculated (F). Differences in partial pairwise correlation coefficients between the 2 groups are indicated and asterisks denote a significant difference (G). The difference maps of “capsaicin versus PBS,” “CSBG versus PBS,” or “CSBG versus capsaicin” show the difference in correlation between the 2 groups for the capsaicin-treated and the PBS-treated groups, the CSBG-treated and the PBS-treated groups, and the CSBG-treated and the capsaicin-treated groups, respectively. Blue color indicates that the former is greater than the latter. Red color indicates that the latter is greater than the former. Error bars, SE; *p < 0.05; **p < 0.01 (two-sample, two-tailed t test).

*C*. *albicans* injected into the hind paw of mice induced pain-related behaviors (Figure S1A). The pain sensation reportedly depends on the direct stimulation of primary sensory neurons by the fungus (Kashem et al., 2015). *C*. *albicans* was found to have already spread its hyphae, as observed in culture, when patients report a pain sensation in the early phase of invasive *Candida* infection (Figure 1A). The hyphae directly damage living cells; however, the molecular mechanisms inducing the pain sensation remain unclear. Recent report suggested that candidalysin, a fungal cytolytic peptide, is released from the hyphae and may evoke calcium influx into the cells (Moyes et al., 2016). Candidalysin induced slight mechanical allodynia; however, allodynia was also induced by the injection of Ece1Δ/Δ *C*. *albicans*, which cannot produce candidalysin, similar to wild-type *C*. *albicans* (Figures S1B–S1D). Furthermore, candidalysin did not induce intracellular calcium increases in the dorsal root ganglion (DRG) neurons isolated from mice (Figure S1E, observations of 43 cells by 3 trials). Therefore, we assumed that there are other molecules causing neural activation followed by uncomfortable sensations. To investigate these molecules, we focused on components of the fungal body and found that β-glucan was secreted from the fungus when cultured for 2 hr at 37°C (Figure 1B). β-Glucan is released as CSBG or *C. albicans*-derived particulate β-glucan (CPBG) in the infected regions (Figure S1F). CSBG might be a particularly important component for the pathological condition because when injected into the hind paw this β-glucan enhanced pro-inflammatory cytokine release, including tumor necrosis factor (TNF)-α, IL-6, and IL-1β, and hind paw enlargement due to the infiltration of myeloid cells in the CSBG-injected area (Figures S1G–S1I). To explore whether the nature of β-glucan-induced pain *in vivo* is similar to TRPV1-mediated pain, fractional amplitude of low-frequency fluctuations (ALFF) analysis of resting-state brain fMRI (Zang et al., 2007, Zou et al., 2008) was performed to quantify the levels of CSBG or capsaicin-induced pain (Figures 1C–1G). The sensation of pain has been associated with activation of the primary somatosensory cortex (S1) and insula (Schweinhardt and Bushnell, 2010). Both CSBG and capsaicin injection significantly increased ALFF in the S1, and CSBG-evoked ALFF was 1.5-times more potent than that evoked by capsaicin (Figure 1F). A significant increase in insula ALFF was observed only in CSBG-treated mice (Figure 1F). Cross-correlation analysis revealed that differences in partial pairwise correlation coefficients between CSBG and capsaicin were significant between the anterior cingulate cortex (ACC) and hippocampus, between the ACC and S1, between the motor area and hippocampus, and between the thalamus and amygdala (Figure 1G). Because these areas are all involved in pain sensation (Bushnell et al., 2013), we conclude that *in vivo* CSBG nociception displays different characteristics in TRPV1-mediated pain.

### CSBG Potently Caused Allodynia Depending on Keratinocyte-Derived ATP

**Figure 2:**
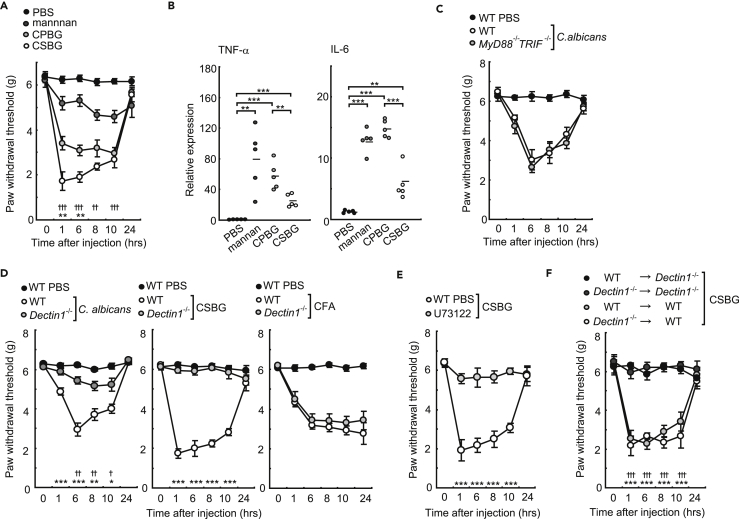
β-Glucan-Induced Allodynia Is Dependent on Dectin-1 (A) Mechanical allodynia in response to *C*. *albicans* components (n = 10/group; ✝, mannan versus phosphate buffered saline (PBS); *CPBG versus CSBG). (B) Mannan, CPBG, or CSBG were injected into the hind paws of mice. After 24 hr, TNF-α and IL-6 expression levels in the hind paws were measured using qPCR (n = 5). (C) *C*. *albicans*-induced mechanical allodynia in *MyD88*^*−/−*^, *TRIF*^*−/−*^, and wild-type (WT) mice (all groups n = 7). (D) Mechanical allodynia with hind paw injection of *C*. *albicans*, CSBG, or CFA in *Dectin1*^*−/−*^ and WT mice (n = 8/group; ✝, WT PBS versus WT; *, WT versus *Dectin1*^*−/−*^). (E) CSBG-induced mechanical allodynia after pretreatment with U73122 (10 μM, 25 μL) (n = 6/group). (F) Mechanical allodynia with hind paw injection of CSBG in WT or *Dectin1*^*−/−*^ mice irradiated and reconstituted with bone marrow from *Dectin1*^*−/−*^ or WT mice (n = 6/group; ✝, WT→*Dectin1*^*−/−*^ versus WT→WT; *Dectin1^*−/−*^→*Dectin1*^*−/−*^ versus *Dectin1*^*−/−*^→WT). Error bars, SE; *or ^✝^ p < 0.05; **or ^✝✝^ p < 0.01; ***or ^✝✝✝^ p < 0.001.

CSBG injection induced inflammatory conditions in hind paws (Figures S1H and S1I). Interestingly, mechanical allodynia was strongly induced by CSBG and CPBG, although mannan also slightly induced allodynia (Figure 2A). The β-glucan- or mannan-induced allodynia recovered after 24 hr, whereas pro-inflammatory cytokine release still occurred (Figures 2A and 2B). Among the *C*. *albicans* components, CSBG is the strongest algesic substance. However, the cytokine stimulatory capacity of CSBG is weak compared with other *C*. *albicans* components (Figure 2B). Moreover, the allodynia induced by *C*. *albicans* was not reduced in *MyD88*- and *TRIF*-deficient mice (Figure 2C). Therefore, inflammatory responses through TLR pathways may not be involved in allodynia. However, Dectin-1 may be an important receptor for mechanical allodynia because *C*. *albicans*- or CSBG-induced allodynia was drastically reduced in Dectin-1-deficient mice, although Complete Freund’s adjuvant (CFA)-induced allodynia was the same between wild-type and Dectin-1-deficient mice (Figure 2D). Notably, CSBG-induced allodynia was suppressed by the PLC inhibitor, U73122 (Figure 2E). Thus, downstream signaling of Dectin-1 activation is important for inducing allodynia.

**Figure 3:**
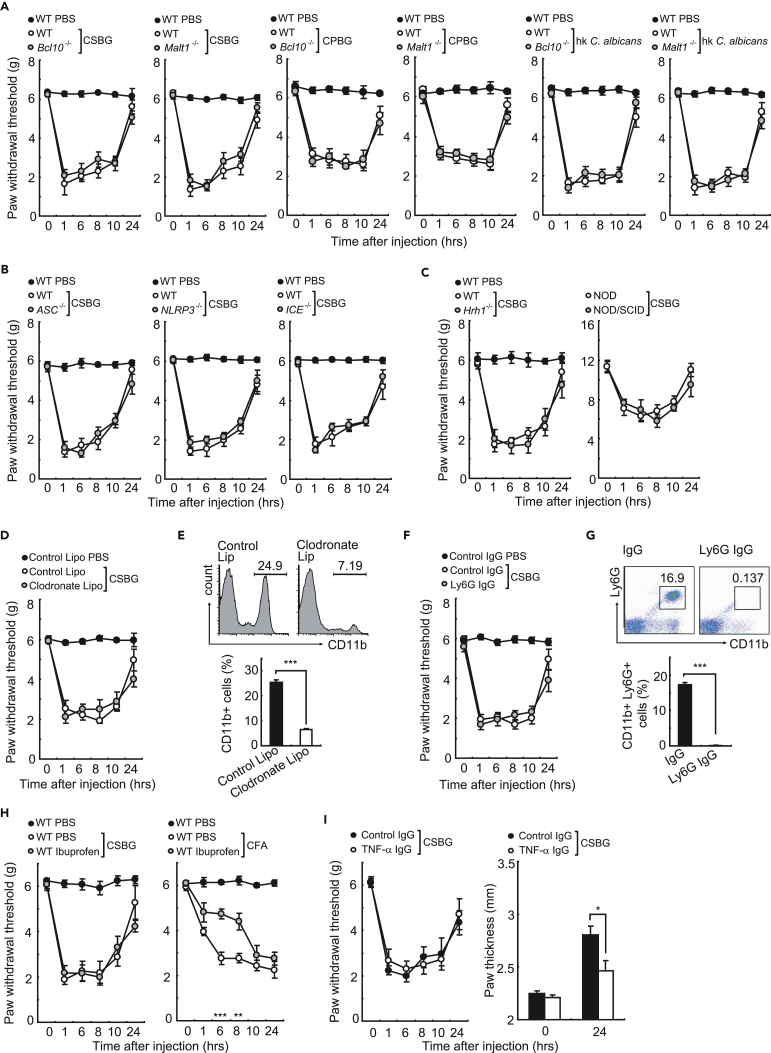
β-Glucan-Induced Allodynia Is Independent of Immunity (A–C) CSBG or heat-killed *C*. *albicans*-induced mechanical allodynia in the indicated mice (A, n = 8; B, n = 10; C, n = 5). (D and E) Clodronate liposome or control liposome were injected into wild-type (WT) mice. After 24 hr, CSBG-induced allodynia was analyzed (D). Cells were prepared from spleens and analyzed by FACS analysis (E) (n = 8). (F and G) Anti-Ly6G antibody or isotype control antibody were injected into WT mice. After 24 hr, CSBG-induced allodynia was analyzed (F). Cells were prepared from spleens and analyzed by FACS (G) (n = 8). (H) Nonselective cyclooxygenase inhibitor, ibuprofen (200 mg/kg), or the vehicle control were orally administered 1 hr before CSBG or CFA injection into the hind paws. Ibuprofen significantly suppressed CFA-induced allodynia, but not CSBG-induced allodynia (n = 8). (I) Anti-TNF-α antibody or isotype control antibody was injected into WT mice. After 24 hr, CSBG-induced allodynia (left) and paw thickness (right) were quantified (n = 5). Error bars, SE; *p < 0.05; ** p < 0.01; ***p < 0.001.

**Figure 4:**
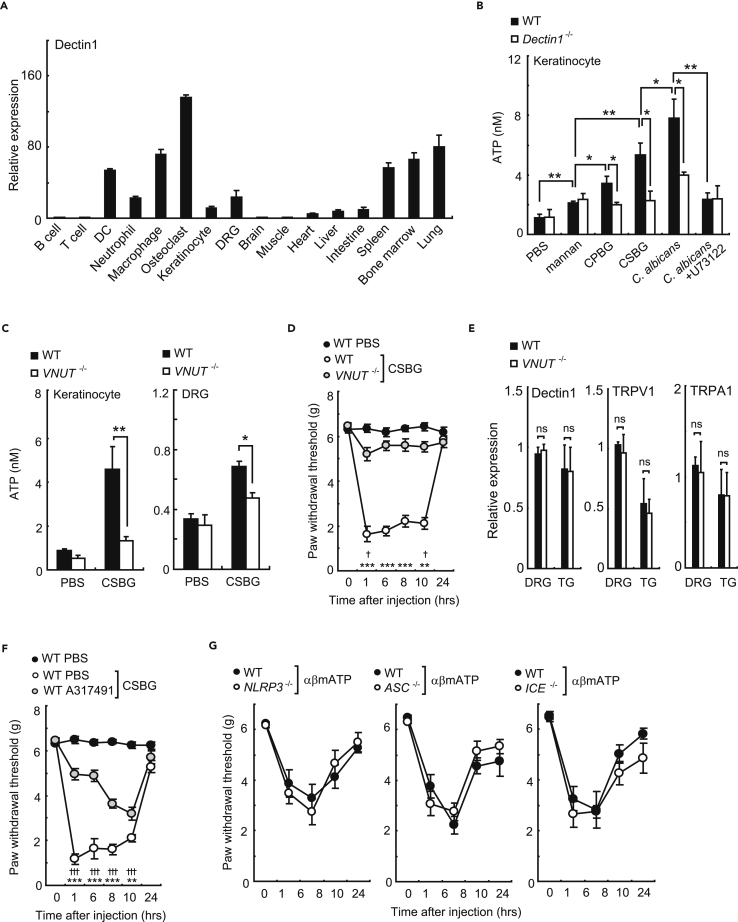
β-Glucan-Induced Allodynia Is Dependent on ATP (A) Expressions of Dectin-1 in various cells and tissues (n = 3). (B) ATP levels in the culture supernatant of keratinocytes stimulated for 3 hr by mannan (100 μg/mL), CSBG (100 μg/mL), CPBG (100 μg/mL), *C*. *albicans*, or *C*. *albicans* plus PLC inhibitor U73122 (10 μM) (n = 4). (C) ATP levels in the culture supernatant of keratinocytes or DRG from *VNUT*^*−/−*^ and WT mice stimulated by CSBG (100 μg/mL) for 3 hr (n = 4). (D) CSBG-induced mechanical allodynia in *VNUT*^*−/−*^ and WT mice (n = 8–18; *VNUT*^*−/−*^ CSBG; ✝, WT phosphate buffered saline (PBS) versus WT; * WT versus *VNUT*^*−/−*^). (E) Dectin-1, TRPV1, and TRPA1 expression levels in the DRG from *VNUT*^*−/−*^ mice and control mice (n = 3). (F) A317491 (10 μM, 25 μL) or PBS were injected into the hind paws of WT mice. After 30 min, CSBG was injected into the hind paws and mechanical allodynia was measured (n = 6/group; ✝, WT PBS versus WT; *, WT versus WT A317491). (G) Mechanical allodynia in αβmATP-treated *NLRP3*^*−/−*^, *ASC*^−/−^, *ICE*^−/−^, and WT mice (n = 6/group). Error bars, SE; *or ^✝^ p < 0.05; **p < 0.01; ***or ^✝✝✝^ p < 0.001.

It has been established that Dectin-1 is mainly expressed in immune cells. Therefore, we investigated the efficacy of the immune system, which may be involved in the pain sensation. Wild-type or Dectin-1-deficient bone marrow engrafted onto Dectin-1-deficient mice showed no mechanical allodynia in response to CSBG, whereas wild-type or Dectin-1-deficient bone marrow-engrafted wild-type mice showed similar allodynia in response to CSBG (Figure 2F). Furthermore, Bcl-10- or Malt-1-deficient strains showed mechanical allodynia similar to wild-type mice in response to CSBG, CPBG, or heat-killed (hk) *C*. *albicans* (Figure 3A). CSBG-induced mechanical allodynia in mice deficient in inflammasome component genes (Figure 3B), histamine receptor H1 (Hrh1)-deficient mice (Figure 3C), non-obese diabetic/severe combined immunodeficiency mice (lacking T and B cells, Figure 3C), clodronate liposome-treated mice (lacking macrophages, Figures 3D and 3E), Ly6G antibody-injected mice (lacking neutrophils, Figures 3F and 3G), ibuprofen-treated mice (Figure 3H), and TNF-α-antibody-injected mice (Figure 3I) was indistinguishable from that in wild-type mice. These results clearly indicated that the immune system is not involved in *C*. *albicans*-induced allodynia, and we hypothesized that a non-hematopoietic cell-derived factor contributes to *C*. *albicans*-induced allodynia. Among the non-immune cells, keratinocytes express Dectin-1 (Figure 4A), and it is reported that an intercellular signal passes from the keratinocytes to neurons via ATP (Mandadi et al., 2009). To test whether *C*. *albicans* induce ATP secretion from keratinocytes, we stimulated keratinocytes with mannan, CPBG, CSBG, and *C*. *albicans*. ATP was strongly released by CPBG, CSBG, and *C*. *albicans* in a Dectin-1-dependent manner, whereas ATP release from isolated DRG neurons was weaker than that from keratinocytes (Figures 4B, 4C, and S2A). To test whether ATP exocytosis is involved in Dectin-1-mediated allodynia, we focused on the VNUT. Keratinocytes isolated from VNUT-deficient mice showed dramatically impaired ATP release in response to CSBG and *C*. *albicans* (Figures 4C and S2B). VNUT is also reportedly expressed in DRG neurons (Nishida et al., 2014), where ATP release was also found to be reduced (Figure 4C). Thus, keratinocytes mainly produce ATP and cell-cell interactions with primary sensory neurons are important for protecting the infected area, although the neurons could be activated by autocrine stimulation. Strikingly, CSBG or hk *C*. *albicans*-induced mechanical allodynia was almost abolished in VNUT-deficient mice, although the expression levels of Dectin-1, TRPV1, and TRPA1 were similar between wild-type and VNUT-deficient mice (Figures 4D, 4E, and S2C). Collectively, our findings provide the first evidence that *C*. *albicans*-induced allodynia is dependent on Dectin-1-stimulated keratinocyte-derived ATP.

It has been reported that cytosolic phospholipase A_2_ (PLA_2_) activation through P2X receptor in DRG neurons is involved in the pathogenesis of neuropathic pain (Tsuda et al., 2007). Therefore, we next investigated the effect of A317491, an inhibitor of P2X_3_ and P2X_2/3_ receptors (Jarvis et al., 2002), on CSBG-induced allodynia. A317491 treatment significantly suppressed CSBG-induced mechanical allodynia (Figure 4F). Recently, it has been reported that ATP activates inflammasomes through P2X receptors (Gombault et al., 2013) and the activated inflammasomes may induce allodynia. Therefore, we investigated the effects of α,β-methylenadenosine 5'-triphosphate (αβmATP) on allodynia in inflammasome-deficient mice, including *NLRP3*, *ASC*, and *ICE* mutant strains. However, αβmATP-induced allodynia was not reduced in these deficient mice (Figure 4G). Thus, we conclude that the pathway to induce allodynia by *C*. *albicans* is molecular signaling between Dectin-1 in keratinocytes and P2X_3_ and P2X_2/3_ in primary sensory neurons.

### Dectin-1-Mediated Activation of TRPV1 and TRPA1 was Critically Involved in CSBG-Induced Pain

Our MRI results suggest that CSBG induced the acute pain sensation (Figure 1). Our previous work suggested that mRNA expression of Dectin-1 is detected in DRG (Maruyama et al., 2017). To check which DRG population expresses Dectin-1, we next analyzed mRNA expression of Dectin-1 in Nav1.8-positive DRG (Figures S3A and S3B). The *Nav1*.*8Cre* mice were coupled with *ROSA26-tdRFP* mice and Nav1.8-positive DRG neurons were separated by fluorescence-activated cell sorting (FACS) analysis depending on the red fluorescent protein (RFP) fluorescence level. Because the Dectin-1 expression level was higher in Nav1.8-positive neurons (Figures S3A and S3B), Dectin-1 appeared to be expressed in Nav1.8-positive primary DRG neurons (Figures S3C–S3E). We next investigated the acute irritant effect of CSBG in Dectin-1-deficient mice (Figure S3F). CSBG-induced pain-related behaviors (cheek wiping) were slightly observed in wild-type mice but not in Dectin1-deficient mice. In contrast, pain-related behaviors were not reduced in Bcl-10- or Malt-1-deficient mice (Figure S3G). Thus, acute pain caused by *C*. *albicans* may depend on Dectin-1 and pro-inflammatory cytokines may be unnecessary for β-glucan-induced acute pain.

Although an extract from hk *C*. *albicans* did not induce potent calcium increases (data not shown), we analyzed CSBG response in DRG neurons from wild-type and Dectin-1-deficient mice. Calcium imaging suggests the functional expression of Dectin-1 in isolated DRG neurons (Figures S3H and S3I). Unexpectedly, 5 of 136 neurons in wild-type mice showed rapid increases in intracellular calcium concentrations that occurred within 1 min after CSBG application (Figure S3G). No rapid calcium increases were detected in Dectin-1-deficient cells (total 64 cells by 6 trials) and vehicle (total 30 cells by 3 trials). We also discovered that DRG neurons express spleen tyrosine kinase (Syk), a critical factor of Dectin-1 signaling, and that CSBG can induce phosphorylation of PLCγ2 in DRG neurons depending on Dectin-1 (Figures S3C and S3J–S3L). Notably, PLC inhibitor U73122 significantly reduced β-glucan-induced pain (Figure 2E). It is thought that TRP channel activation could also be accelerated by the PLA_2_-PKC axis (Nishizuka, 1992) and TRPV1 and TRPA1 activations downstream of GPCR depend on PLCβ activation, and these pathways are crucial in inflammatory conditions induced by factors such as bradykinin (McMahon and Wood, 2006). However, our novel finding suggests the importance of phosphorylated PLCγ2 in DRG neurons on inflammatory conditions induced by β-glucan. These results indicate that some DRG neurons could be directly involved in CSBG detection. However, acute pain-related behaviors were induced by hk *C*. *albicans* injection (Figure S4). Furthermore, the behaviors were reduced in TRPV1- and/or TRPA1-deficient mice and inhibited by U73122 treatment. Thus, the relationships between keratinocytes and DRG neurons could be important in *in vivo* situation, although *in vivo* ATP releases could not be analyzed.

**Figure 5:**
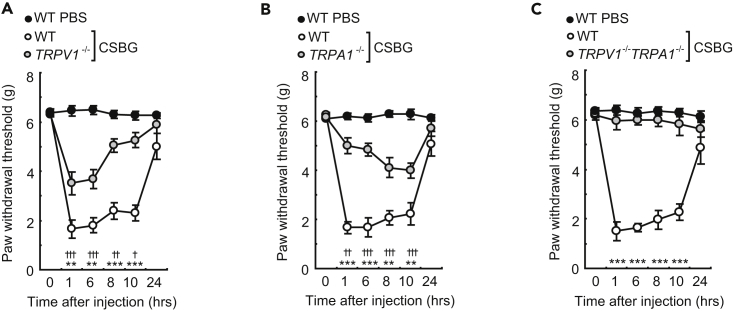
TRPV1 and TRPA1 Were Indispensable for β-Glucan-Induced Allodynia (A–C) Mechanical allodynia following hind paw injection with CSBG in (A) *TRPV1*^*−/−*^, (B) *TRPA1*^*−/−*^, and (C) *TRPV1*^*−/−*^*TRPA1*^*−/−*^ mice (n = 6–8/group; ✝, WT phosphate buffered saline (PBS) versus WT; *, WT versus mutant) (n = 10/group). Error bars, SE; ^✝^ p < 0.05; ** or ^✝✝^ p < 0.01; *** or ^✝✝✝^ p < 0.001.

Next, we investigated whether TRPV1 and TRPA1 were involved in CSBG-induced allodynia; TRPV1- or TRPA1-deficient mice showed incomplete reduction of mechanical allodynia, which was completely inhibited in TRPV1/TRPA1 double-deficient mice (Figures 5A–5C). Strikingly, hk *C*. *albicans*-induced acute pain-related behaviors were also inhibited in mice deficient in TRPV1 and/or TRPA1 (Figure S4). Thus, these results indicate that the Dectin-1-mediated activation of TRPV1 and TRPA1 in primary sensory neurons is an important signaling pathway in β-glucan-induced pain sensation in *Candida* infection.

### Clodronate Inhibits CSBG-Mediated Enhancement of Histamine-Independent Itch Sensation and CSBG-Induced Mechanical Allodynia

**Figure 6:**
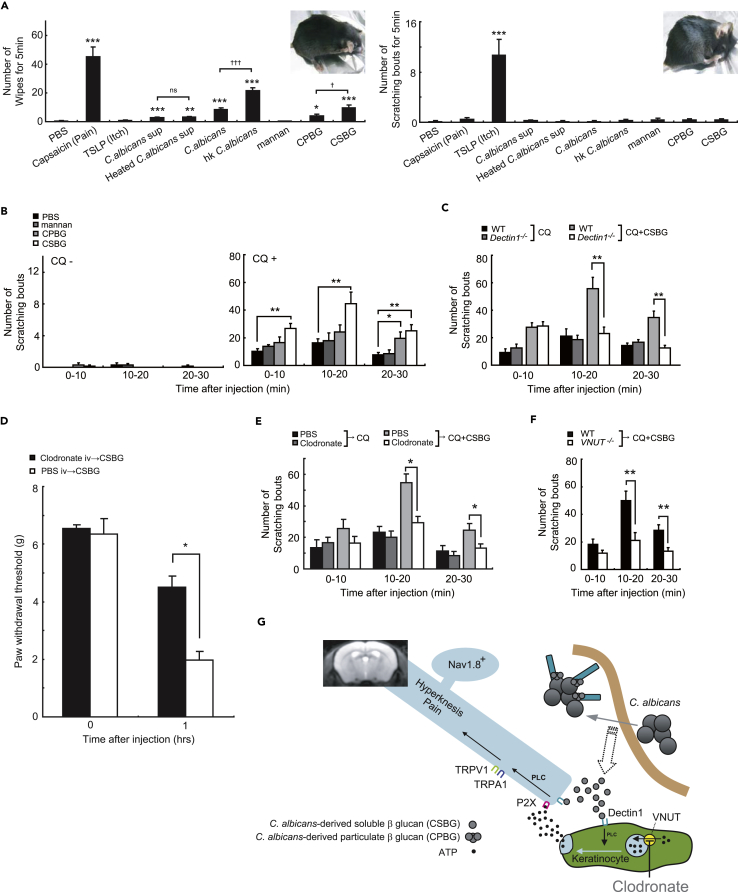
β-glucan-Induced Allodynia and Enhancement of Histamine-Independent Itch were Neutralized by VNUT Inhibitor Clodronate (A) Wiping and scratching behaviors after injection of mice with *C*. *albicans* or its components (15 μg, n = 10 for each injection group). (B) Mannan, CPBG, or CSBG were injected into the cheek with or without chloroquine (CQ), and scratching behaviors were observed (n = 7 for each injection group). (C) CQ was injected into the cheek of *Dectin1*^*−/−*^ or WT mice with or without CSBG, and scratching behaviors were observed (n = 7 for each injection group). (D) CSBG-induced mechanical allodynia after preinjection with clodronate (10mg/kg intravenous [IV] injection, 60 min before CSBG injection into footpad. n = 6/group; *, phosphate buffered saline (PBS) IV→CSBG versus clodronate→CSBG). (E) PBS or clodronate (300μg/25ul) was injected into the cheek. After 60 min, CQ was injected into the cheek with or without CSBG, and scratching behaviors were observed (n = 7 for each injection group). (F) CQ plus CSBG was injected into the cheek of *VNUT*^*−/−*^ or WT mice, and scratching behaviors were observed (n = 7 for each injection group). (G) Model for the novel innate sensory mechanisms of *Candida* infection. *C*. *albicans*-derived β-glucan directly stimulates Nav1.8-positive pain nerve via Dectin-1 to induce acute pain. *C*. *albicans*-derived β-glucan also induces allodynia and hyperknesis (abnormal pruriceptive state in which there is a normally pruritic stimulus). Notably, β-glucan-induced allodynia was not dependent on the immune system, but instead on keratinocyte-derived ATP. Error bars, SE; *or ^✝^ p < 0.05; **p < 0.01; *** or ^✝✝✝^ p < 0.001.

In a clinical situation, *Candida* infection causes an itch sensation. To clarify the pruritogenic effects of *C*. *albicans in vivo*, we investigated whether *C*. *albicans* triggers acute pain-related behaviors (wiping) with or without itching using a cheek injection model (Figure 6A). *C*. *albicans* was observed to cause wiping behaviors, and this response was significantly enhanced by heat killing. The *C*. *albicans* culture supernatant also caused wiping behaviors, and this effect was not changed by heating of the supernatant. Scratching behaviors were never observed, indicating that the acute phase of infection with *C*. *albicans* may cause pain rather than itching. Therefore, we hypothesized that β-glucan enhances the itch sensation induced by the pruritogen CQ, because TRPA1 is reportedly involved in cell signaling of the itch sensation, whereas TRPV1 is not required in this histamine-independent itch pathway (Wilson et al., 2011). Interestingly, CSBG and CPBG significantly enhanced CQ-induced itch-related behaviors and these behaviors were inhibited in Dectin-1-deficient mice (Figures 6B and 6C). Recently, it has been reported that clodronate is a strong inhibitor of VNUT (Kato et al., 2017). A low concentration of clodronate impaired vesicular ATP release from cells. To check the prophylactic potential of clodronate to β-glucan-mediated mechanical allodynia and enhancement of histamine-independent itch sensation, we intravenously or subcutaneously injected clodronate into mice 60 min before the ligand injection. To our surprise, clodronate pretreatment significantly inhibited β-glucan-induced mechanical allodynia and β-glucan-mediated enhancement of CQ-induced itch behavior (Figures 6D and 6E). Strikingly, CSBG-plus-CQ-induced itch-related behaviors were dramatically impaired in VNUT-deficient mice (Figure 6F). Collectively, these findings clearly indicate that Dectin-1 and VNUT are crucial components to express neural functions involved in both pain and itch sensations evoked by β-glucan and clodronate can be used to treat unpleasant feelings induced by fungal infection (Figure 6G).

## Discussion

Here we showed that CSBG is a potent irritant secreted by *C*. *albicans.* A previous report suggested that MyD88 signaling mediates Dectin-1 ligand zymosan-induced allodynia accompanied by the production of pro-inflammatory cytokines such as TNF-α and IL-1β (Guerrero et al., 2012). Contrary to this report, our *in vivo* analysis revealed that innate immunity has limited effects on CSBG-induced allodynia. Discrepancies between our findings and this previous report may be explained by the difference in ligand composition (zymosan is composed of mannan plus β-glucan). Significantly, no allodynia was detected when CSBG was injected into the hind paw of VNUT-deficient mice, which cannot secrete ATP in response to Dectin-1 stimulation. A recent report suggested that clodronate inhibits VNUT at a half maximal inhibitory concentration of 15.6 nM without affecting other vesicular transporters, acting as an allosteric modulator through competition with Cl^−^ (Kato et al., 2017). Our *in vivo* behavioral assay clearly indicated that low-dose clodronate treatment can be used to abolish the unpleasant feelings induced by β-glucan. Intriguingly, systemic injection of large-dose clodronate liposome to deplete macropahges had no impact on β-glucan-induced allodynia. Such discrepancy may be explained by the severe cytotoxicity of high-concentration clodronate liposome. Notably, inflammasome components were dispensable for αβmATP-induced allodynia, suggesting that ATP signaling in nociception does not use the inflammasome cascade. Recently, it has been reported that nociceptors directly sense *Staphylococcus aureus* cytolytic components such as α-hemolysin (Chiu et al., 2013). In contrast to *S*. *aureus*, *C*. *albicans*-derived newly discovered cytolytic peptide candidalysin did not contribute to enhance the intracellular calcium concentrations in DRG. In contrast, infection with candidalysin-deficient *C*. *albicans* revealed a slight impairment in allodynia compared with wild-type *C*. *albicans*. Thus, candidalysin may induce cytotoxicity in keratinocytes, leading to an increase in extracellular ATP, which weakly contributes to allodynia, and to our knowledge, this is the first study to provide evidence that extracellular ATP evokes fungal allodynia. A recent study in mice revealed that repeated vaginal *C*. *albicans* infections cause mechanical allodynia accompanied by mucosal hyperinnervation with nociceptors (Farmer et al., 2011). This study also reported that vaginal mechanical allodynia can persist long after the resolution of active *C*. *albicans* infection. Vulvar pain associated with previous infection (vulvodynia) affects large numbers of women of childbearing age (Farmer et al., 2011). Because the most promising treatment of severe vulvodynia is surgical excision of the vulval tissue (Goldstein and Burrows, 2008), ATP- or VNUT-targeted therapy such as clodronate treatment may be a promising drug repositioning.

In this study, we also showed that CSBG activates Nav1.8-positive DRG neurons via Dectin-1 to evoke acute pain. A previous *in vitro* study suggested that hk *C*. *albicans* induces a calcium influx in TRPV1-positive nociceptors (Kashem et al., 2015). Contrary to this study, cross-correlation analysis of resting-state fMRI clearly indicated that *in vivo* CSBG nociception displays different characteristics in TRPV1-mediated pain. In myeloid cells, Dectin-1 signaling is mainly activated by CPBG, which triggers phagocytosis, leading to the clustering of Dectin-1 receptors in synapse-like structures, from which the regulatory tyrosine phosphatases CD45 and CD148 are excluded (Goodridge et al., 2011). Our results indicated that the algesic activity of CPBG was weaker than that of CSBG. Because DRG neurons do not express CD45 or CD148 and are not considered to be phagocytic, Dectin-1 signaling in DRG neurons may behave differently compared with that in myeloid cells. Our behavioral analysis further suggested that *C*. *albicans*-induced acute pain is dependent on the Dectin-1-mediated activation of the PLC-TRPV1/TRPA1 axis. In this study, we also showed the synergic effect of Dectin-1 signaling on CQ-mediated itch behaviors. Dectin-1 signaling may activate TRPA1, which could be the mechanism that causes the vulvovaginal candidiasis-induced itch.

Collectively, *C*. *albicans* stimulates Nav1.8-positive nociceptors via the Dectin-1 to induce acute pain. *C*. *albicans*-derived β-glucan induces allodynia, which depends on the ATP transporter VNUT, but not on the immune system. This suggests that fungal allodynia induction requires extracellular ATP. Our previous study revealed that hind paw β-glucan injection after nociceptor ablation or in TRPV1/TRPA1 deficiency showed dramatically increased osteoinflammation accompanied by impaired CGRP production (Maruyama et al., 2017). Notably, CGRP inhibited β-glucan-induced cytokine production and bone resorption by osteoclasts. These previous discoveries and the findings of this study clearly indicate that the signaling pathway from Dectin-1 to TRP channels is a novel molecular mechanism of pain generation and that CGRP production accelerated by sensory nervous excitation is critical for the resolution of fungal inflammation.

### Limitations of the Study

It would be intriguing to analyze keratinocyte- or DRG-specific Dectin-1- (or VNUT-) deficient mice. To address such an important issue as future direction is important.

## Methods

All methods can be found in the accompanying Transparent Methods supplemental file.

## Supplemental Information

Document S1. Transparent Methods and Figures S1–S4
