# Supplementary material for: The ATP Transporter VNUT Mediates Induction of Dectin-1-Triggered Candida Nociception
Source: iScience. 2018 Aug 15;6:306–18. doi: 10.1016/j.isci.2018.08.007 (PMC6137711; doi:10.1016/j.isci.2018.08.007)
Supplement: Document S1. Transparent Methods and Figures S1–S4 [file mmc1.pdf]

ISCI, Volume *6*

Supplemental Information

The ATP Transporter VNUT Mediates Induction of Dectin-1-Triggered *Candida* Nociception

Kenta Maruyama, Yasunori Takayama, Erika Sugisawa, Yu Yamanoi, Takashi Yokawa, Takeshi Kondo, Ken-ichi Ishibashi, Bikash Ranjan Sahoo, Naoki Takemura, Yuki Mori, Hisashi Kanemaru, Yutaro Kumagai, Mikaël M. Martino, Yoshichika Yoshioka, Hisao Nishijo, Hiroki Tanaka, Atsushi Sasaki, Naohito Ohno, Yoichiro Iwakura, Yoshinori Moriyama, Masatoshi Nomura, Shizuo Akira, and Makoto Tominaga

Supplemental Information

The ATP transporter VNUT mediates induction of Dectin-1-triggered *Candida* nociception

Kenta Maruyama, Yasunori Takayama, Erika Sugisawa, Yu Yamanoi, Takashi Yokawa, Takeshi Kondo, Ken-ichi Ishibashi, Bikash Ranjan Sahoo, Naoki Takemura, Yuki Mori, Hisashi Kanemaru, Yutaro Kumagai, Mikaël M. Martino, Yoshichika Yoshioka, Hisao Nishijo, Hiroki Tanaka, Atsushi Sasaki, Naohito Ohno, Yoichiro Iwakura, Yoshinori Moriyama, Masatoshi Nomura, Shizuo Akira and Makoto Tominaga

Kenta Maruyama and Yasunori Takayama contributed equally to this work.

Figure S1

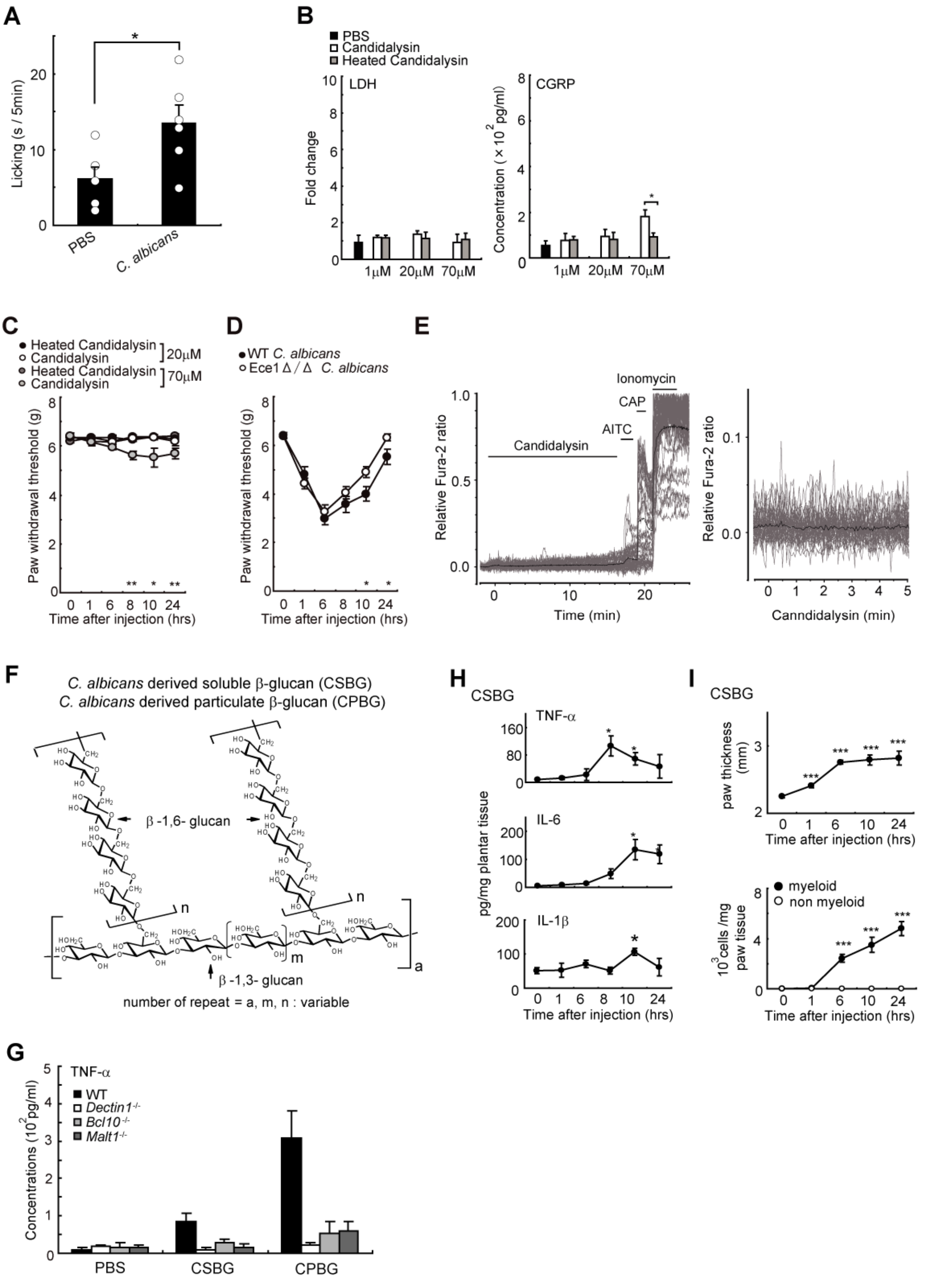

Figure S1. *C. albicans* and its components induce pain-related behaviors and inflammation.

Related to Fig. 1, Figure 2 and Figure 3. (A) *C. albicans*–induced pain-related licking behaviors (n=6).

(B) LDH release (left) or CGRP production (right) from DRG neurons treated with 70 μM candidalysin for 24 h (n=3). (C) Candidalysin and heated candidalysin (20 μM or 70 μM) were injected into the hind paws of WT mice and mechanical allodynia was analyzed (n=10; *, heated candidalysin 70 μM vs. candidalysin 70 μM). (D) WT *C. albicans* or Ece1Δ/Δ *C. albicans* were injected into the hind paws of WT mice and mechanical allodynia (n=10) was analyzed. (E) Relative Fura-2 ratio with candidalysin (left), and expanded traces (right). Black lines, average value of relative Fura-2 ratio; gray lines, response of each neuron (n=43). (F) Structure of CPBG and CSBG. *C. albicans*–derived β-glucan is made up of long branched 1,6-β-glucan and 1,3-β-glucan segments. (G, H) TNF-α production by CSBG or CPBG stimulated macrophages (G) and various cytokine levels in CSBG-injected hind paw tissues measured by ELISA (n=8). (I)Paw thickness and myeloid or lymphoid cell numbers in CSBG-injected hind paw tissues were measured (n=8). Error bars, S.E; * $p < 0.05$; *** $p < 0.001$.

Figure S2

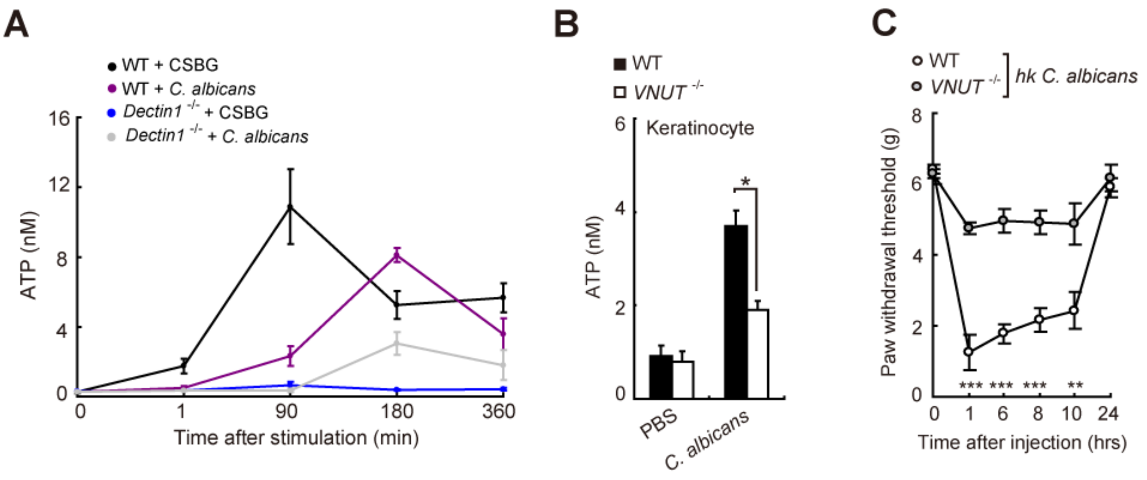

Figure S2. VNUT is critical for *C. albicans*-induced pain. Related to Figure 4. (A) ATP levels in the culture supernatant of keratinocytes stimulated by CSBG (100 μg/ml) or *C. albicans* (n=4). (B) ATP levels in the culture supernatant of keratinocytes from *VNUT*$^{-/-}$ and WT mice stimulated by *C. albicans* for 2 h (n=4). (C) hk *C.albicans*-induced mechanical allodynia in *VNUT*$^{-/-}$ and WT mice (n=7). Error bars, S.E; * $p < 0.05$; ** $p < 0.01$; *** $p < 0.001$.

Figure S3

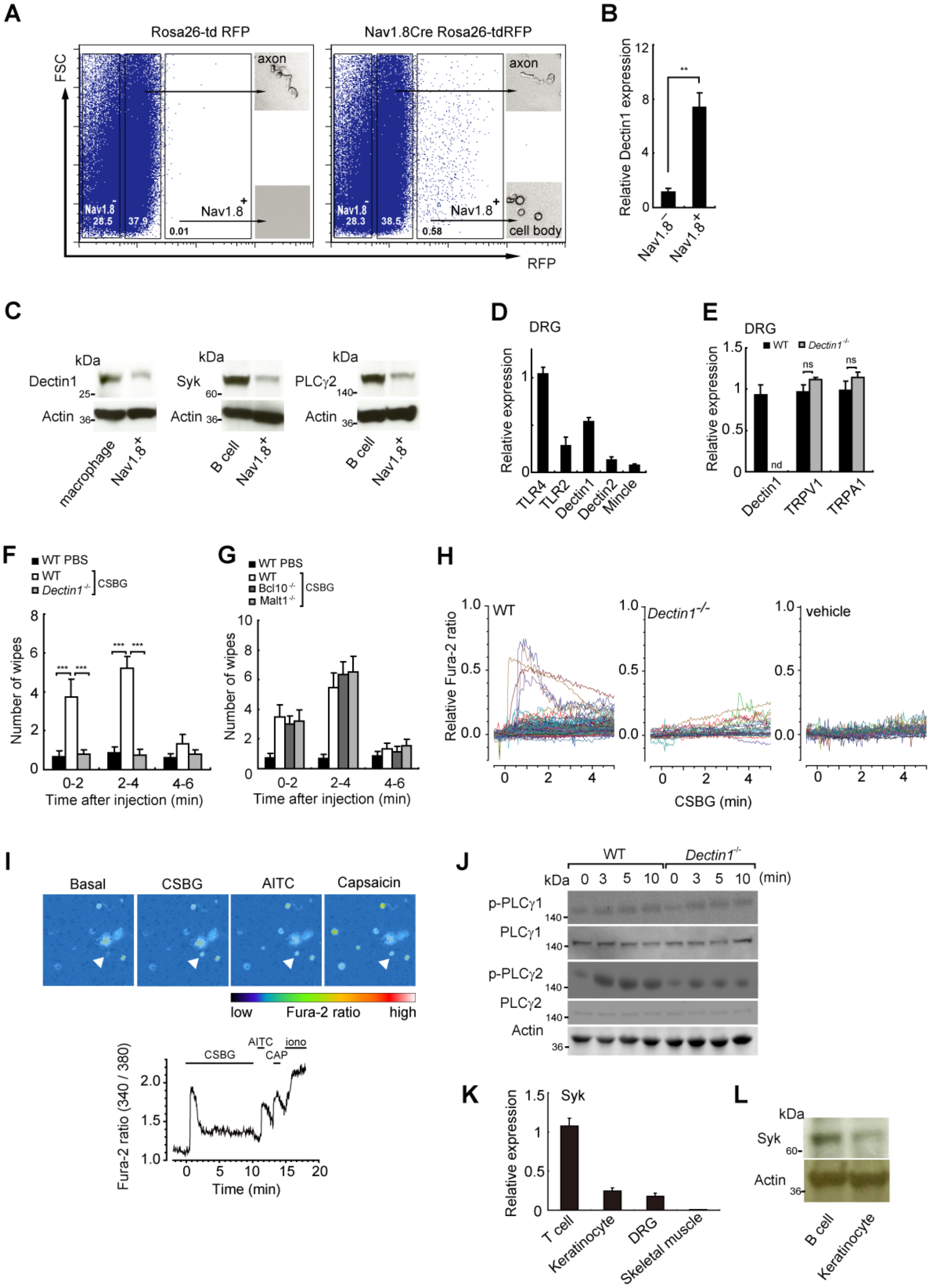

Figure S3. Direct activation of nociceptor by Dectin-1. Related to Figure 1 and Figure 5. (A) DRG from *Rosa26-tdRFP* and *Nav1.8Cre Rosa26-tdRFP* mice was dissociated and analyzed using

FACSAria. (B) Nav1.8$^+$ neurons (RFP$^+$) and Nav1.8- neurons (RFP$^-$) were sorted from the DRG of *Nav1.8Cre Rosa26-tdRFP* mice. Dectin-1 and CGRP expression levels in both populations were measured by qPCR (n=3). (C) Nav1.8$^+$ neurons (RFP$^+$ Nav1.8$^+$ area cells in A) were sorted from the DRG of *Nav1.8Cre Rosa26-tdRFP* mice. Dectin1, Syk and PLCγ2 expression levels were analyzed by western blotting. (D) DRG fungal receptor expression (n=3). (E) DRG Dectin-1 and TRP channel expression (n=3). (F) Wiping behaviors after cheek injection of CSBG into *Dectin1*$^{-/-}$and WT mice (n=7–15). (G) Wiping behaviors after cheek injection of CSBG into *Bcl10*$^{-/-}$, *Malt1*$^{-/-}$, and WT mice (n=7–15). (H) Intracellular calcium levels in hk *C. albicans*–treated DRG neurons. The Fura-2 ratio is relative to the peak ionomycin response. (I) All traces of relative Fura-2 ratio with 100 µg/mL CSBG or the vehicle control in DRG neurons. (J) Phosphorylated PLCγ1 and PLCγ2 levels in DRG neurons from *Dectin1*$^{-/-}$ or WT mice stimulated by CSBG for the indicated times. (K) Expression of Syk in indicated cells (n=3). (L) Syk expression in keratinocyte was analyzed by western blotting. Error bars, S.E.; * $p < 0.05$; ** $p < 0.01$; *** $p < 0.001$.

Figure S4

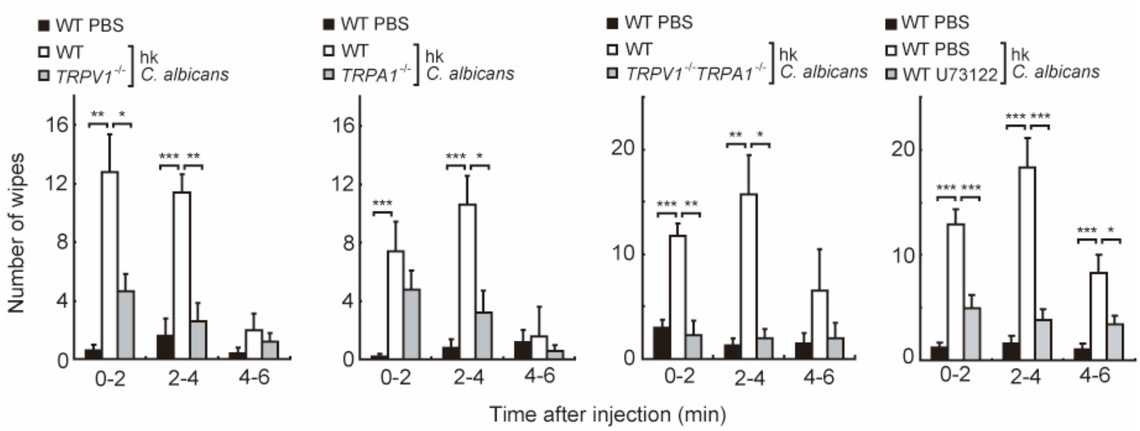

Figure S4. ***C. albicans*-induced nociception is mediated by the PLC-TRPV1/TRPA1 axis.** Related to Figure 1 and Figure 5. Wiping behaviors after cheek injection with hk *C. albicans* (into *TRPV1*$^{-/-}$ mice, *TRPA1*$^{-/-}$ mice, *TRPV1*$^{-/-}$ *TRPA1*$^{-/-}$ mice, PLC inhibitor [U73122]-treated WT mice, and WT mice [n=5–10]). Error bars, S.E.; * $p < 0.05$; ** $p < 0.01$; *** $p < 0.001$; ns, not significant.

Transparent Methods

Mice

For infection or behavioral experiments, 8- to 13-week-old female mice were used. *MyD88*$^{-/-}$ *TRIF*$^{-/-}$ mice (Adachi et al., 1998; Yamamoto et al., 2003), *Hrh1*$^{-/-}$ mice (Inoue et al., 1996), *TRPV1*$^{-/-}$ mice (Caterina et al., 2000), *TRPA1*$^{-/-}$ mice (original strain was backcrossed for more than eight generations with C57BL/6) (Bautista et al., 2006), and *VNUT*$^{-/-}$ mice (Sakamoto et al., 2014) were generated and control C57BL/6 mice were purchased from SLC or Japan CLEA. NOD/ShiJic-scidJcl (NOD SCID) mice (Koyanagi et al., 1997) and NOD wild-type mice were purchased from Japan CLER. *Dectin1*$^{-/-}$ mice (Saijo et al., 2007), *NLRP3*$^{-/-}$ mice (Mariathasan et al., 2006), *ASC*$^{-/-}$ mice (Mariathasan et al., 2004), and *ICE*$^{-/-}$ mice (Gu et al., 1997) were generated as previously described and were compared with WT littermate controls. *Bcl-10*$^{-/-}$ mice and *Malt-1*$^{-/-}$ mice were a gift from S. Gerondakis, S. Morris, and V.M. Dixit, respectively (Ruefli-Brasse et al., 2003; Xue et al., 2003) and were compared with WT littermate controls (in Fig 2K, control WT mice were purchased from Oriental Bio Service). *Rosa26-tdRFP* mice were generated as described previously (Luche et al., 2007). *Nav1.8-Cre* mice (B6;129-Scn10atm(cre)Jnw/H, stock id EM:04582) were purchased from the European Mouse Mutant Archive (EMMA). Animal experiments complied with the institutional animal care and use guidelines of Osaka University (animal 14013) and the National Institutes of Natural Sciences (16A074).

Pathogens and their components

C. albicans strain THK519, designated *C. albicans*, was obtained from a patient admitted to Tohoku University Hospital. *C. albicans*–derived soluble β-glucan (CSBG) and *C. albicans*–derived particulate β-glucan (CPBG) were prepared from *C. albicans* strain NRBC1385 (same as ATCC18804, ATCC20308, CBS562, JCM1542, and NRRL Y-12983), purchased from the NITE Biological Resource Center (Chiba, Japan) as previously described (Ishibashi et al., 2002). To generate *C. albicans*–derived β-glucan, acetone-dried *C. albicans* NRBC1385 was suspended in 0.1 M NaOH with NaClO (final concentration, 1%) for 1 day at 4°C. After the reaction was completed, the reaction

mixture was centrifuged and the particulate fraction was collected and dried by ethanol and acetone. The dried particulate fraction was suspended in saline and sonicated for 30 s. After centrifugation, the supernatant was removed and the particulate fraction was designated CPBG (CPBG is insoluble to saline). To prepare CSBG, CPBG suspended in DMSO was ultrasonically disrupted and the resulting supernatant was designated as CSBG. To generate the vehicle control of CSBG, saline suspended CPBG was centrifuged and the resulting supernatant was designated as "vehicle". To evaluate the purity of CSBG, elemental analysis and ^{13}C-NMR spectrum analysis was performed. Assignment of ^{13}C-NMR Chemical shifts clearly indicates that our CSBG contains only β-1,3/β-1,6-glucan peaks (data not shown). *C. albicans* ECE1 homozygous knockout mutant strain (M2057, designated Ece1Δ/Δ *C. albicans*) and the wild-type control strain (M1477, designated WT *C. albicans*) were provided by Dr. B. Hube (Moyes et al., 2016). Yeast from fungal strains was cultured on potato dextrose agar plates (KOHJIN BIO, Saitama, Japan) at 30°C. After a 30-h incubation, cells were harvested, suspended in PBS, and counted using a hemocytometer and trypan blue staining. *C. albicans*–derived purified mannan, designated mannan, was obtained from the National Institute for Biological Standards and Control (NIBSC code 76/515, non WHO reference material). *C. albicans*–derived peptide Ece1-III K 62-92K (SIIGIIMGILGNIPQVIQIIMSIVKAFKGNK), designated here as candidalysin, was synthesized by GenScript (Lot No:16017867190002/PE2773, Purity 98.4%). *C. albicans* was heat killed at 56°C for 30 min. To generate hyphae from fungi, yeast from *C. albicans* was cultured in RPMI (Wako, Osaka, Japan) supplemented with 10% FCS for 2 h at 37°C. The β-glucan concentration in the fungal culture supernatant was quantified using Fungitec G test MKII Nissui (Nissui, Tokyo, Japan). To prepare *C. albicans* culture supernatant, *C. albicans* was cultured in RPMI supplemented with 10% FCS for 30 h at 37°C and centrifuged to collect the supernatant. Additional information on the fungal and bacterial strains, and the cells and reagents used, is provided in the Supplemental Information.

In vivo injection of pathogens and their components

Injection into the hind paws or cheeks was performed using a 30-gauge needle (Becton Dickinson).

For fungal infection, 25 μl of the live or hk yeast form of the fungi in PBS was injected subcutaneously into the hind paws (3×10^5 c.f.u.) or cheeks (3×10^7 c.f.u.) and behavioral analysis was performed. For dead bacterial administration, 25 μl of hk *S. aureus* (3×10^8 c.f.u.) in PBS was injected subcutaneously into the cheeks of mice. CSBG, CPBG, or mannan were subcutaneously injected into the hind paws or cheeks (15 μg per 25 μl PBS into hind paws and 150 μg per 25 μl PBS into cheeks). Capsaicin (40 μg per 25 μl PBS, M2028, Sigma-Aldrich, USA), TSLP (3 μg per 25 μl PBS, MAB555, R&D, USA), and chloroquine (200 μg per 25 μl PBS, C6628, Sigma-Aldrich, USA) were subcutaneously injected into the cheeks of mice. To test the algesic effect of ATP, α,β-methylene ATP (100 nM, 25 ml, 5.05419.0001, Calbiochem, USA) was subcutaneously injected into the hind paws. After injection, mice were monitored and assayed for pain and/or itch behaviors and tissue inflammation.

Behavioral analysis

Mechanical sensitivity was measured using polypropylene tips attached to Von Fray apparatus (electronic Von Frey anesthesiometer, IITC Inc., Woodland Hills, CA, USA), as described previously (Zylka et al., 2008). Briefly, mice were acclimated to the testing room, equipment, and experimenter for 12–36 h before behavioral testing. Mice were tested in a resting state. The experimenter was blinded to the genotype during testing, and was trained to apply the polypropylene tip to the central hind paw with a gradual increase in pressure. A mirror placed below the grid provided guidance for the tip to the hind paw. To test the wiping and scratching behaviors, mice were placed in separate box (15 × 15 × 20 cm) and observed for 30 min, as previously described (Shimada and LaMotte, 2008).

Scanning electron microscopic analysis

Scanning electron microscopic analysis of *C. albicans* was performed, and samples were fixed with 3% formaldehyde in 0.1 M phosphate buffer (pH 7.4), and post-fixed with 1% osmium tetroxide and 0.8% potassium ferrocyanide in the same buffer. Next, samples were treated with 1% tannic acid solution, washed with water, and then fixed with 0.5% osmium tetroxide and 0.5% potassium ferrocyanide in 0.1 M phosphate buffer (pH 7.4). Samples were then dehydrated in graded ethanol

concentrations ending with 100% ethanol and dried using a critical point drier. Samples were coated with osmium tetroxide vapor using a glow discharging device and observed using an S-4800 FE-scanning electron microscope (Hitachi High-Technologies Corp., Japan) at 1.5 kV and a 2 mm working distance.

MRI analysis

Structural T1-weighted, resting-state fMRI data were collected using a Varian 4.7T scanner (Unity INOVA, Varian Associates, Palo Alto, CA, USA) with a JASTEC Horizontal Magnet 4.7T (JMTB-4.7/310/SS, Japan Superconductor Technology, Kobe, Japan) with a 72-mm volume coil and a two-channel phased array mouse brain coil (Rapid Biomedical, Rimpar, Germany). Before each structural T1-weighted, resting-state fMRI scanning session, an exogenous contrast agent, USPIO (Molday ION Carboxl, CL30Q02-7, Funakoshi, Japan) was injected into the caudal vein (12–15 mg/kg) to optimize the localization of fMRI signals (Leite and Mandeville, 2006). After placing the animal in a cradle, mice were maintained under light anesthesia with 1.5% isoflurane and then, CSBG, capsaicin or the PBS control were subcutaneously injected into the right hind paw (15 μg per 25 μl PBS). Anatomic images were obtained using a gradient-echo technique (GE3D; data matrix, 128 × 128 × 64; FOV, 2.0 cm^3; TR/TE, 0.02/0.008 s; flip angle, 10°). From these images, nine 1-mm thick planes with no gaps were selected for functional imaging. In all scans, T2*-weighted functional MR images were obtained using a gradient-echo imaging technique (data matrix, 32 × 32; FOV, 2.5 cm^2; TR/TE, 0.01/0.006 s; flip angle, 8°; slice t thickness, 1 mm; scan time, 0.32 s/slice × 9 slices + 0.12 s delay=3.0 s). After data collection, normalization to standard space and smoothing was performed. Registration of the fMRI space data to standard space was carried out using bioimagesuite35 (http://bioimagesuite.yale.edu). After manual registration, an affine transformation with 12 degrees of freedom was performed. The standard space was chosen to be an anatomical scan from a rat of size 128 × 128 × 55 mm^3 with a voxel size of 0.156 × 0.156 × 0.31 mm^3. Finally, 3D smoothing with a 0.8 mm size filter was carried out. To visualize the pain status in the brain, ALFF was calculated using the Resting-State fMRI Data Analysis Toolkit V1.8 REST (http://restfmri.net/forum/index.php). For each

voxel, a discrete Fourier transform was performed on the resting-state time series. The ALFF was computed by measuring the average square-root of the total power spectrum from 0.01–0.1 Hz on a voxel-by-voxel basis. Cross-correlation region-of-interest analysis and second level analysis were performed by UF2C (version 6.2), a plug-in of spm8 (version 6313), to make individual or group cross-correlation maps. A pairwise correlation was calculated between each pair of regions-of-interest, using our own MATLAB software. To compare the differences in the correlation values among the three groups, UF2C was also used to carry out second-level analysis. Seeds of the cross-correlation were supplied as a coordinates list. The shape of the seeds was a cube with four voxels per edge.

Generation of bone marrow chimeric mice

Donor bone marrow cells from *Dectin-1*$^{-/-}$ and age-matched control wild-type mice were collected and 1×10^7 cells were intravenously injected into lethally-irradiated 4-week-old recipient mice. Mice were analyzed at 7 weeks after bone marrow transplantation.

PCR and protein analysis

RNA was extracted using TRIzol (Invitrogen Life Science Technologies). Reverse transcription was performed using ReverTra Ace (Toyobo Co. Ltd.). The quantity of mRNA was normalized to 18S rRNA using the TaqMan ribosomal control reagent kit (Applied Biosystems). Western blotting was performed as previously described (Maruyama et al., 2012). Immunohistochemistry of DRG and trigeminal ganglion (TG) is described in Supplemental Information.

DRG neuron isolation

The DRGs were separated from L1–L6 in mice after perfusion with 10 mL of ice-cold artificial cerebrospinal fluid (aCSF; 124 mM NaCl, 5 mM KCl, 1.2 mM KH_2PO_4, 1.3 mM $MgSO_4$, 2.4 mM $CaCl_2$, 10 mM glucose, 24 mM $NaHCO_3$, equilibrated with 95% O_2 and 5% CO_2 for 1 h on ice). The tissues were incubated with 725 µg collagenase type IX (lot# SLBG3258V and SLBG3259V, Sigma-Aldrich) in 250 µL of Earle's balanced salt solution (Sigma-Aldrich) at 37°C for 25 min. Next, DRG

neurons were mechanically separated by 10–20 cycles of pipetting using a small diameter Pasteur pipette. The neurons were centrifuged three times at 300 × *g* for 5 min at 4°C. Then, supernatants were discarded and fresh PBS or aCSF was added to wash out the collagenase. The isolated neurons were placed on 12-mm-diameter coverslips (Matsunami, Japan) with 40 μL of aCSF and were maintained at room temperature in a 95% O_2 and 5% CO_2 humidified chamber.

Cells and reagents

B cells and T cells were prepared from splenocytes using anti-B220 and anti-Thy-1.2 magnetic beads (Miltenyi Biotec, Bergisch Gladbach, Germany), respectively. Splenic dendritic cells were prepared using anti-CD11c magnetic beads (Miltenyi Biotec). Splenic $CD11b^+$ macrophages were sorted by FACSAria (BD Biosciences, Bedford, MA, USA). Antibodies for FACS analysis were purchased from BD Biosciences, and cells were stained with the indicated antibodies. Data were acquired using FACSCalibur (BD Biosciences), and analyzed by FlowJo software (Ashland, OR, USA). RFP-positive DRG neurons from *Nav1.8Cre Rosa26-tdRFP* mice were sorted using FACSAria. Primary keratinocytes were obtained from newborn wild-type mice as previously described (Sokabe et al., 2010). Neutrophils were harvested from the peritoneal cavity of mice 2 h after intra-peritoneal injection of thioglycollate. Macrophage colony stimulating factor (M-CSF)-derived macrophages and conventional dendritic cells were generated by bone marrow cell cultures with M-CSF and GM-CSF, respectively. Keratinocytes (2×10^5) or DRGs were cultured with various stimuli, and ATP levels in the supernatant were quantified using an ATP Colorimetric/Fluorimetric Assay Kit (#K354-100, BioVision, USA). Cell death was quantified using an LDH Cytotoxicity Colorimetric Assay Kit (#K311-400, Bio Vision). Western blotting was performed as previously described (Maruyama et al., 2012). Proteins from DRG, Nav1.8 positive neuron and keratinocyte were detected using anti-Dectin1 (MAB1859, R&D Systems), anti-Syk (#2712, Cell Signaling), anti-PLCγ1 (EPR5358, ab109501), anti-PLCγ2 (#3872, Cell Signaling), anti-p-PLCγ1 (2821S, Cell Signaling), anti-p-PLCγ2 (3874S, Cell Signaling) and anti-actin (C-11, Santa Cruz Biotechnology).

Calcium imaging

Isolated DRG neurons were cultured at 37°C for 1 h in Earle's balanced salt solution (Sigma-Aldrich) with Fura-2 AM (Molecular Probes). Fura-2 fluorescence of isolated mouse DRG neurons was measured in a standard bath solution: 140 mM NaCl, 5 mM KCl, 2 mM $MgCl_2$, 2 mM $CaCl_2$, 10 mM glucose, 10 mM HEPES, pH 7.40 with NaOH, excited at wavelengths of 340 and 380 nm, and emission was monitored at 510 nm with a CCD camera (CoolSnap ES; Roper Scientific/Photometrics) or a sCMOS camera (Zyla DG-152XC1E-F1; ANDOR Technology). Data were acquired and analyzed by IPlab (Scananalytics), iQ (ANDOR), and ImageJ software. Ionomycin (5 µM) was applied to confirm cell viability.

In vivo injection of antibodies and chemicals

To deplete Ly6G-positive neutrophils, anti-Ly6G antibody (250 µg/head, RB6-8C5, eBioscience, USA) or isotype control antibody were intraperitoneally injected 24 h before CSBG injection into the hind paws of mice. To deplete T cells, anti-CD3 antibody (200 µg/head, 17A2, eBioscience) or isotype control antibody were intraperitoneally injected 12 h before CSBG injection into the hind paws of mice. To neutralize TNF-α, anti-TNF-α antibody (200 µg/head, TN3-19.12, BioLegend, USA) or isotype control antibody were intraperitoneally injected 12 h before CSBG injection into the hind paws of mice. To deplete macrophages, chlodronate liposome (200 µl/head, HYGIEIA Bioscience, Osaka, Japan) or control liposome were intraperitoneally injected 24 h before CSBG injection into the hind paws of mice. To inhibit cyclooxygenase pathway, nonselective cyclooxygenase inhibitor ibuprofen (200 mg/kg) or the vehicle control were orally administered using disposable animal feeding needles (FUCHIGAMI, cat no. 6202) 1 h before CSBG or CFA injection into the hind paws of mice. For PLC or ATP receptor inhibition, U73122 (10 µM, 25 µl, U6756, Sigma-Aldrich) or A317491 (10 µM, 25 µl, A2979, Sigma-Aldrich) were injected subcutaneously into both hind paws 30 min before ligand injection, respectively. For VNUT inhibition, Disodium Clodronate Tetrahydrate (D4160, TOKYO CHEMICAL INDUSTRY) was injected intravenously or subcutaneously 1h before ligand injection. For paw thickness evaluation, a digital micrometer (CD67-S PM, Mitutoyo, Kanagawa, Japan) was

used.

Statistical analysis

The difference in mean values between two groups was compared using Student's *t*-test, with $p < 0.05$ considered to be significant.

References

Adachi, O., Kawai, T., Takeda, K., Matsumoto, M., Tsutsui, H., Sakagami, M., Nakanishi, K., and Akira, S. (1998). Targeted disruption of the MyD88 gene results in loss of IL-1- and IL-18-mediated function. Immunity *9*, 143-150.

Bautista, D.M., Jordt, S.E., Nikai, T., Tsuruda, P.R., Read, A.J., Poblete, J., Yamoah, E.N., Basbaum, A.I., and Julius, D. (2006). TRPA1 mediates the inflammatory actions of environmental irritants and proalgesic agents. Cell *124*, 1269-1282.

Caterina, M.J., Leffler, A., Malmberg, A.B., Martin, W.J., Trafton, J., Petersen-Zeitz, K.R., Koltzenburg, M., Basbaum, A.I., and Julius, D. (2000). Impaired nociception and pain sensation in mice lacking the capsaicin receptor. Science *288*, 306-313.

Gu, Y., Kuida, K., Tsutsui, H., Ku, G., Hsiao, K., Fleming, M.A., Hayashi, N., Higashino, K., Okamura, H., Nakanishi, K., *et al.* (1997). Activation of interferon-gamma inducing factor mediated by interleukin-1 beta converting enzyme. Science *275*, 206-209.

Inoue, I., Yanai, K., Kitamura, D., Taniuchi, I., Kobayashi, T., Niimura, K., Watanabe, T., and Watanabe, T. (1996). Impaired locomotor activity and exploratory behavior in mice lacking histamine H-1 receptors. Proceedings of the National Academy of Sciences of the United States of America *93*, 13316-13320.

Ishibashi, K., Miura, N.N., Adachi, Y., Ogura, N., Tamura, H., Tanaka, S., and Ohno, N. (2002). Relationship between the physical properties of Candida albicans cell well beta-glucan and activation of leukocytes in vitro. International Immunopharmacology *2*, 1109-1122.

Koyanagi, Y., Tanaka, Y., Kira, J., Ito, M., Hioki, K., Misawa, N., Kawano, Y., Yamasaki, K., Tanaka, R., Suzuki, Y., *et al.* (1997). Primary human immunodeficiency virus type 1 viremia and central nervous system invasion in a novel hu-PBL-immunodeficient mouse strain. Journal of Virology *71*, 2417-2424.

Leite, F.P., and Mandeville, J.B. (2006). Characterization of event-related designs using BOLD and IRON fMRI. Neuroimage *29*, 901-909.

Luche, H., Weber, O., Rao, T.N., Blum, C., and Fehling, H.J. (2007). Faithful activation of an extra-bright red fluorescent protein in "knock-in" Cre-reporter mice ideally suited for lineage tracing studies. European Journal of Immunology *37*, 43-53.

Mariathasan, S., Newton, K., Monack, D.M., Vucic, D., French, D.M., Lee, W.P., Roose-Girma, M., Erickson, S., and Dixit, V.M. (2004). Differential activation of the inflammasome by caspase-1 adaptors ASC and Ipaf. Nature *430*, 213-218.

Mariathasan, S., Weiss, D.S., Newton, K., McBride, J., O'Rourke, K., Roose-Girma, M., Lee, W.P., Weinrauch, Y., Monack, D.M., and Dixit, V.M. (2006). Cryopyrin activates the inflammasome in response to toxins and ATP. Nature *440*, 228-232.

Maruyama, K., Kawagoe, T., Kondo, T., Akira, S., and Takeuchi, O. (2012). TRAF Family Member-associated NF-kappa B Activator (TANK) Is a Negative Regulator of Osteoclastogenesis and Bone Formation. Journal of Biological Chemistry *287*, 29114-29124.

Moyes, D.L., Wilson, D., Richardson, J.P., Mogavero, S., Tang, S.X., Wernecke, J., Hofs, S., Gratacap, R.L., Robbins, J., Runglall, M., *et al.* (2016). Candidalysin is a fungal peptide toxin critical for mucosal infection. Nature *532*, 64-68.

Ruefli-Brasse, A.A., French, D.M., and Dixit, V.M. (2003). Regulation of NF-kappa B-dependent lymphocyte activation and development by paracaspase. Science *302*, 1581-1584.

Saijo, S., Fujikado, N., Furuta, T., Chung, S.H., Kotaki, H., Seki, K., Sudo, K., Akira, S., Adachi, Y., Ohno, N., *et al.* (2007). Dectin-1 is required for host defense against Pneumocystis carinii but not against Candida albicans. Nature Immunology *8*, 39-46.

Sakamoto, S., Miyaji, T., Hiasa, M., Ichikawa, R., Uematsu, A., Iwatsuki, K., Shibata, A., Uneyama, H., Takayanagi, R., Yamamoto, A., *et al.* (2014). Impairment of vesicular ATP release affects glucose metabolism and increases insulin sensitivity. Scientific Reports *4*.

Shimada, S.G., and LaMotte, R.H. (2008). Behavioral differentiation between itch and pain in mouse. Pain *139*, 681-687.

Sokabe, T., Fukumi-Tominaga, T., Yonemura, S., Mizuno, A., and Tominaga, M. (2010). The TRPV4 Channel Contributes to Intercellular Junction Formation in Keratinocytes. Journal of Biological Chemistry *285*, 18749-18758.

Xue, L.Q., Morris, S.W., Orihuela, C., Tuomanen, E., Cui, X.L., Wen, R.R., and Wang, D.M. (2003). Defective development and function of follicular, marginal zone, and B1B cells in Bcl10-deficient mice. Blood *102*, 197b-197b.

Yamamoto, M., Sato, S., Hemmi, H., Hoshino, K., Kaisho, T., Sanjo, H., Takeuchi, O., Sugiyama, M.,

Okabe, M., Takeda, K., *et al.* (2003). Role of adaptor TRIF in the MyD88-independent toll-like receptor signaling pathway. Science *301*, 640-643.
Zylka, M.J., Sowa, N.A., Taylor-Blake, B., Twomey, M.A., Herrala, A., Voikar, V., and Vihko, P. (2008). Prostatic Acid Phosphatase Is an Ectonucleotidase and Suppresses Pain by Generating Adenosine. Neuron *60*, 111-122.
